# Eyes-Open and Eyes-Closed Resting State Network Connectivity Differences

**DOI:** 10.3390/brainsci13010122

**Published:** 2023-01-10

**Authors:** Junrong Han, Liwei Zhou, Hang Wu, Yujuan Huang, Mincong Qiu, Likai Huang, Chia Lee, Timothy Joseph Lane, Pengmin Qin

**Affiliations:** 1 Key Laboratory of Brain, Cognition and Education Science, Ministry of Education, Institute for Brain Research and Rehabilitation, Guangdong Key Laboratory of Mental Health and Cognitive Science, South China Normal University, Guangzhou 510631, China; 2 Center for Studies of Psychological Application, School of Psychology, South China Normal University, Guangzhou 510631, China; 3 Department of Neurology, TMU Shuang Ho Hospital, New Taipei City 235041, Taiwan; 4 Brain and Consciousness Research Centre, TMU Shuang Ho Hospital, New Taipei City 235041, Taiwan; 5 Graduate Institute of Mind, Brain and Consciousness, Taipei Medical University, Taipei 106052, Taiwan; 6 Institute of European and American Studies, Academia Sinica, Taipei 115201, Taiwan; 7 Pazhou Lab, Guangzhou 510335, China

**Keywords:** resting state network, eyes open, eyes closed, salience network, fMRI

## Abstract

Resting state networks comprise several brain regions that exhibit complex patterns of interaction. Switching from eyes closed (EC) to eyes open (EO) during the resting state modifies these patterns of connectivity, but precisely how these change remains unclear. Here we use functional magnetic resonance imaging to scan healthy participants in two resting conditions (viz., EC and EO). Seven resting state networks were chosen for this study: salience network (SN), default mode network (DMN), central executive network (CEN), dorsal attention network (DAN), visual network (VN), motor network (MN) and auditory network (AN). We performed functional connectivity (FC) analysis for each network, comparing the FC maps for both EC and EO. Our results show increased connectivity between most networks during EC relative to EO, thereby suggesting enhanced integration during EC and greater modularity or specialization during EO. Among these networks, SN is distinctive: during the transition from EO to EC it evinces increased connectivity with DMN and decreased connectivity with VN. This change might imply that SN functions in a manner analogous to a circuit switch, modulating resting state relations with DMN and VN, when transitioning between EO and EC.

## 1. Introduction

The resting state—spontaneous, intrinsic activity—is a fundamental state of human brains [1]. Operationally, it involves those states wherein participants neither respond to external stimuli nor engage in any tasks; instead, their minds wander [2]. It has become an essential paradigm for the investigation of the brain activity of diverse populations [3].

The resting state may be scanned under varying conditions, including eyes closed, EC, or eyes open, EO [4]. The switch from EO to EC appears to modify functional connectivity (FC) among brain regions, resulting in higher connectivity among specific regions [5,6]. In addition, topological organization of networks also exhibit more connectivity among certain regions, correlating with the transition from EO to EC [7]. These findings revealed that the number and the strength of connectivity among multiple regions increased in EC but decreased in EO.

Moreover, the intra-network and internetwork FC between the nodes of resting state networks (RSNs) would be affected by EO and EC. For example, previous studies found decreased intra-network FC in the default mode network, DMN [8] and increased intra-network FC in the auditory network, AN, during the shift from EO to EC [9]. Multiple lines of investigation show that the internetwork FC among nodes of RSNs are modulated by EC and EO. Included among the results of these investigations: during EC elevated FC was evinced between the lingual gyrus (LING) of the visual network (VN) and the superior temple gyrus (STG) of the auditory network (AN) [6], between the posterior cingulate cortex (PCC) of the DMN and the STG of the AN [10], as well as between the PCC of the DMN and the anterior insula (AI) of the salience network (SN) [10]. However, previous studies have also indicated that there remains substantial controversy about the differential connectivity among RSNs, in particular for the VN and other associated networks [4,9]. In short, EC-EO differential patterns of connectivity among RSNs remain unclear.

To investigate connectivity difference among RSNs, seven RSNs were included in the present study: (1) The SN, comprising the anterior insula (AI) and the anterior cingulate cortex (ACC) [11] is involved in detecting salient stimuli, modulating network activity, attention and working memory [12]. (2) The DMN, comprising the medial prefrontal cortex (MPFC), the posterior cingulate cortex (PCC) and the inferior parietal cortex (IPL) is engaged when task-performance is not required [13]. (3) The central executive network (CEN), comprising the dorsolateral prefrontal cortex (DLPFC) and posterior parietal cortex, is involved in external guided awareness [14]. (4) The dorsal attention network (DAN), comprising the intraparietal sulcus (IPS) and frontal eye field (FEF), is involved in top-down orienting and attention [14]. In addition, three sensory networks—the motor network (MN), the AN and the VN—were also considered in our study.

The aim of the present study is to test the hypothesis that EC, relative to EO, elicits more and stronger patterns of FC among RSNs. In addition, we devoted special attention to the hypothesis that the SN plays a distinctive role in modulating the switch between externally- and internally-oriented neural activity [15]. Toward these ends, we scanned healthy subjects in the two specified states, eyes closed and open. We then performed voxel-wise FC to identify the RSNs and compared EC to EO.

## 2. Materials and Methods

Two datasets were collected separately in different sites. The first dataset was acquired at Free University of Berlin and the second at Taipei Medical University. These two datasets are combined together for the following FC analysis.

### 2.1. Dataset 1 Berlin

#### 2.1.1. Participants

This dataset served as the basis for an investigation that has already been published [16]. Eighteen healthy volunteers participated in this study (15 female; mean age = 27.1; age range = 20–34 years). These subjects did not suffer from any medical, neurological, or psychiatric disorders. Informed written consent was obtained from each. The study was approved by the ethics committee of the Free University of Berlin and the data had been used in a previous study [16]. Two subjects exhibited head movement of greater than 3 mm and another one did not complete the experiment. Therefore, 15 subjects were included in the analysis.

#### 2.1.2. MRI Data Acquisition

Images were acquired on a Siemens 3.0T MAGNETOM TrioTim syngo MRI scanner at the Free University of Berlin. A 3D anatomical image was first acquired using a fast SPGR sequence (TR = 1.9 ms, TE = 2.25 ms, FOV = 256 mm × 256 mm, matrix = 256 × 256, slice thickness = 1 mm) for functional image registration and localization. After that, EC/EO sequences were obtained from each participant; the order of EC/EO runs was counterbalanced across participants.

During both scans, participants were instructed to avoid head movement and to avoid thinking about specific events or concerns, or at least avoid dwelling on any particular thing. In other words, let their minds wander [17]. Participants were also instructed to remain awake; subsequently, they were asked as to whether they had fallen asleep. During the EO run, participants were asked to keep their eyes open and fixate on a white cross displayed on a black background on the in-scanner screen. During the EC run, participants were instructed to close their eyes. All participants reported that they had remained awake throughout the scanning session.

Blood oxygen-level dependent (BOLD) images were acquired using an EPI sequence (TR = 2 s, TE = 30 ms, flip angle = 90°, FOV = 192 mm × 192 mm, matrix = 64 × 64, slice thickness = 3 mm, gap = 0 mm, 37 slices) and 205 volumes were acquired in each run (7 min).

### 2.2. Dataset 2 New Taipei City

#### 2.2.1. Participants

This dataset also served as the basis for an investigation that has already been published [18]. Seventeen healthy volunteers took part in this study (9 female; mean age = 31.9; age range = 23–58 years). None of the participants had a history of neurological or psychiatric disorders. All participants gave their written, informed consent and were compensated financially for their time. The study was approved by the Taipei Medical University Institutional Review Board (TMU-JIRB 201411043). All MRI scanning was carried out during morning sessions at Taipei Medical University’s Shuang Ho Hospital, coordinated by the hospital’s Brain and Consciousness Research Centre.

#### 2.2.2. MRI Data Acquisition

MR images were acquired on a GE MR750 3 Tesla scanner using a standard 8-channel head coil. A high-resolution T1-weighted anatomical image was acquired first. Following this, BOLD imaging was carried out, with the order of these scans counterbalanced across participants. The EO/EC sequence was also counterbalanced across participants and the light in the scanner room was turned off while scanning. As was the case with the Berlin dataset, during both types of scans, participants were instructed to lie still, remain awake and not focus their attention on anything in particular.

For EC runs, participants kept their eyes closed throughout and a black screen was presented to ensure that their exposure to light was minimal. During EO runs, a gray screen was presented. For EO runs, participants were instructed to keep their eyes open and gaze at the screen. After scanning, participants were asked whether they had remained awake; all reported that they had done so.

BOLD-sensitive images were acquired using a T2-weighted EPI sequence (TR = 2000 ms; TE = 30 ms; flip angle = 90°; FOV = 220 mm; matrix = 64 × 64; slice thickness = 3.4 mm; slice gap = 0 mm; 33 slices). 200 volumes were acquired in each run (6.67 min).

#### 2.2.3. Data Preprocessing

All MRI images were processed using the AFNI software package [19]. First, the initial three volumes were excluded; this was followed by spike removal and slice time correction. All functional images were registered to the base volume, which has minimal outliers. Then the anatomical images were skull stripped and aligned to functional images. Next, the anatomical and functional images were converted to MNI space via non-linear transformation and the spatial resolution of functional images were resampled to 3 mm^3^. Subsequently, the functional images were smoothed using 6 mm at the full-width half-maximum (FWHM) Gaussian kernel. Each voxel of data was passed through a band-pass filter (0.01–0.1 Hz), regressed out the mean time course within white matter and head motion parameters (i.e., six demeaned motion parameters and first differences of six motion parameter) to reduce nuisance effect. Furthermore, motion was quantified as the Euclidean norm calculated from the six motion parameters for two consecutive TRs. A motion displacement of >0.2 mm was considered excessive. The volume, as well as the one preceding, that corresponded to excessive motion were removed. Volumes for which 10% of voxels exceeded the base volume were also excluded.

#### 2.2.4. Functional Connectivity Analysis

**Figure 1:**
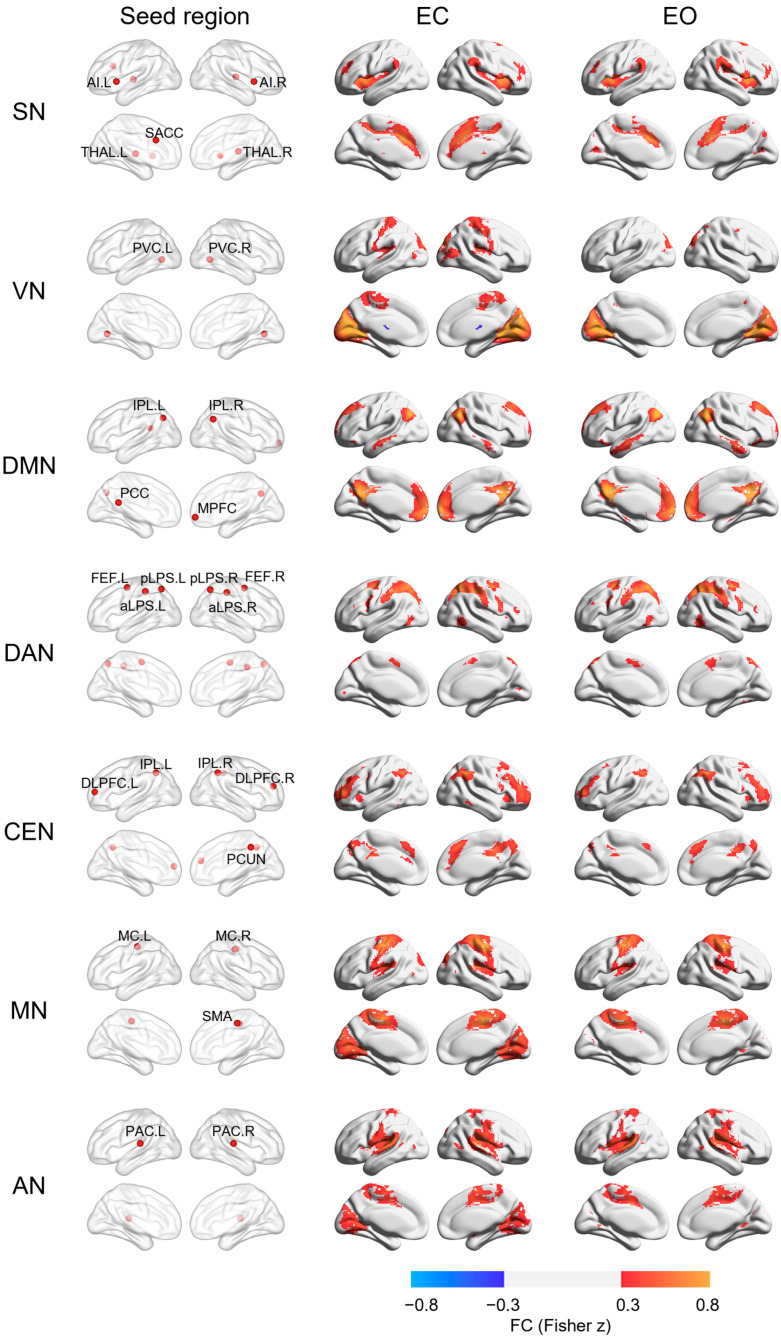
Seed regions of seven networks and their FC maps in EC and EO conditions. The left column shows the seed regions selected for each network. The middle and right column display the FC maps of each network in EC and EO conditions, respectively. The color range of FC maps represents the Fisher z value from −0.8 to −0.3 and from 0.3 to 0.8. SACC = supra-genual anterior cingulate cortex; AI = anterior insula; THAL = thalamus; PCC = posterior cingulate cortex; MPFC = medial prefrontal cortex; PVC = primary visual cortex; IPL = inferior parietal cortex; FEF = frontal eye field; pIPS = posterior intraparietal sulcus; aIPS = anterior intraparietal sulcus; DLPFC = bilateral dorsal lateral prefrontal cortex; PCUN = precuneus; MC = motor cortex; SMA = supplemental motor area; PAC = primary auditory cortex; L = left; R = right.

In order to locate seven networks in both resting conditions, 27 spherical regions (6 mm radius) representing these resting networks were chosen. For SN, five spherical regions with a radius of 6 mm were selected: supra-genual anterior cingulate cortex (SACC), bilateral anterior insula (AI) and bilateral THAL [20]. For VN, bilateral primary visual cortex (PVC) was chosen [21]. For DMN, four regions: PCC, MPFC, bilateral IPL, were chosen as representative [20]; for DAN, six regions: bilateral posterior intraparietal sulcus (pIPS), bilateral anterior intraparietal sulcus (aLPS) and bilateral front eye field (FEF) [21]; for CEN, five regions: bilateral dorsal lateral prefrontal cortex (DLPFC), bilateral inferior parietal lobe (IPL) and precuneus (PCUN) [20]; for MN, three regions: bilateral motor cortex (MC) and supplemental motor area (SMA) [22]; and for AN: bilateral primary auditory cortex (PAC) [22]. Coordinates of seed regions are shown in Table 1 and Figure 1.

For each network, the selected regions were combined and the mean BOLD time series was extracted for voxel-wise functional connectivity analysis. Then, using Pearson correlation analysis, the mean BOLD time series from the combined regions was correlated to other voxels within the whole brain. Next, using the Fisher Z transformation, correlation r values were transformed into z values.

One sample t-test was performed separately for the FC map of each network during EC and EO conditions, to verify that the components of each network corresponded to the targeted network. For each participant, six head motion parameters were demeaned and their average difference between TRs was calculated. Then these six motion differences, gender, age and the site of the scanner were taken as the covariates in the one sample *t*-test. To display the targeted networks clearly, the threshold of Fisher z value was set >0.3 and <−0.3 and the multiple correction was conducted using 3dClustsim with cluster-wise alpha < 0.05. These analyses were performed on the combined dataset and each separate dataset. BrainNet Viewer was used on these FC maps in order to visualize them [23].

Furthermore, for each network, a paired *t*-test was used to compare the FC maps for EC and EO conditions. The covariates used in the one sample t-test were also included in this paired *t*-test. The voxel-wise significance of paired t-tests were *p* < 0.005; they were multi-compare corrected in cluster-wise alpha < 0.05 with 3dClustSim in AFNI. In addition, we overlapped the automated anatomical labeling atlas (AAL3) [24] over the clusters that showing significant difference in the paired t-test, to identify the brain regions of these clusters.

Finally, we constructed seven matrices to show the network-wise FC difference between two conditions for seven networks. Specifically, we first constructed seven network templates by combining the brain regions of AAL3 that belong to the same network [25]. For example, the template of SN was made up of SACC, THAL and insula (INS); VN template was composed of LING, cuneus (CUN) and calcarine fissure (CAL); DMN template consisted of PCC, IPL and MPFC, in which MPFC was made by combing medial superior frontal gyrus (SFGmedial) and medial orbital superior frontal gyrus (PFCventmed); DAN template was made with middle frontal gyrus (MFG) and IPS, in which IPS was made by combing IPL and superior parietal lobe (SPL); CEN template included dorsolateral superior frontal gyrus (SFGdor), PCUN and IPL; MN template included precentral gyrus (PreCG), postcentral gyrus (PoCG) and SMA; AN template included Heschl’s gyrus (HES) and STG. Then we overlapped these templates on the result maps from the paired t-test of each network. The regions contained in the same network were grouped together and their mean FCs were calculated for both resting conditions. Next, the difference between FCs for the two conditions within the grouped regions were compared using paired *t*-test and FDR correction was conducted for multiple comparisons. Accordingly, we constructed seven network-based matrices (7 × 7). These matrices exhibited the FC difference of each network vis-à-vis the other six networks, thereby vividly depicting the alteration of the correlations among networks.

## 3. Results

**Figure A1:**
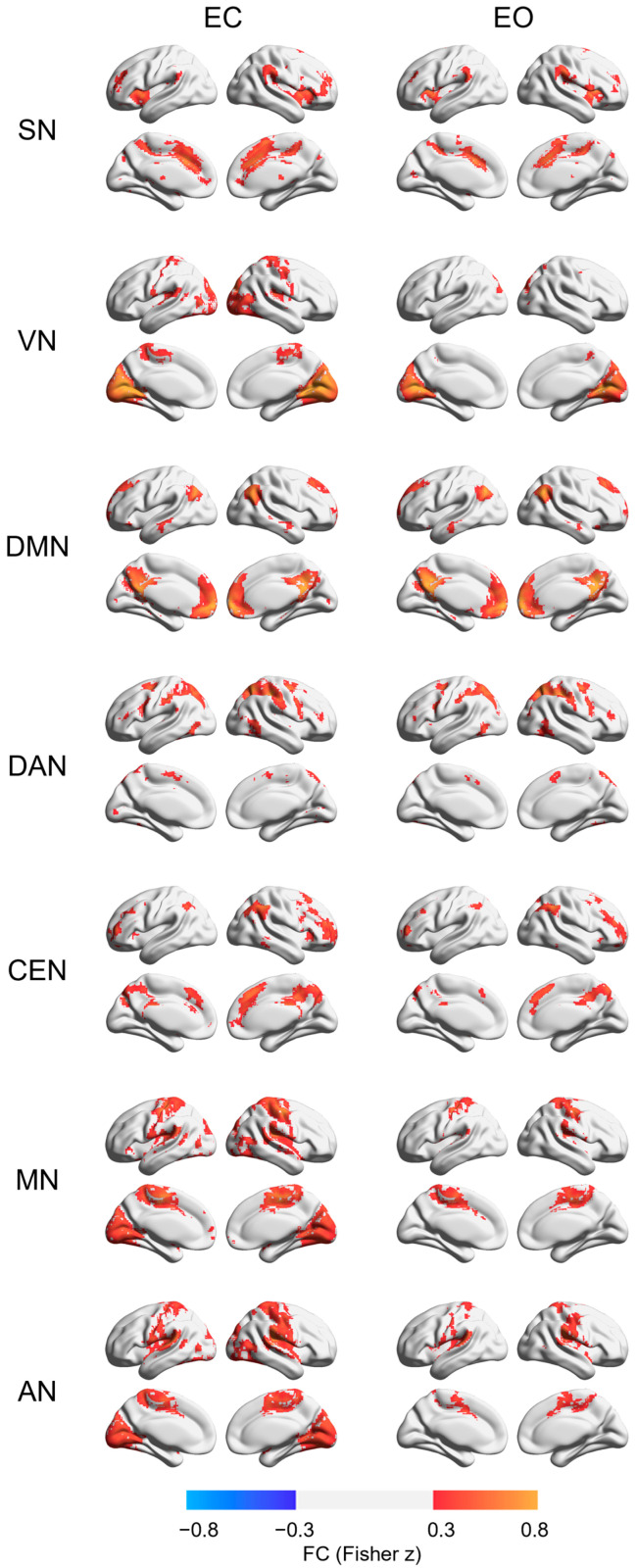
Seven FC maps of networks in EC and EO conditions based on Berlin dataset. The left and right column displays the FC maps of each network in EC and EO conditions, respectively. The color range of FC maps represents the Fisher z value from −0.8 to −0.3 and from 0.3 to 0.8.

**Figure A2:**
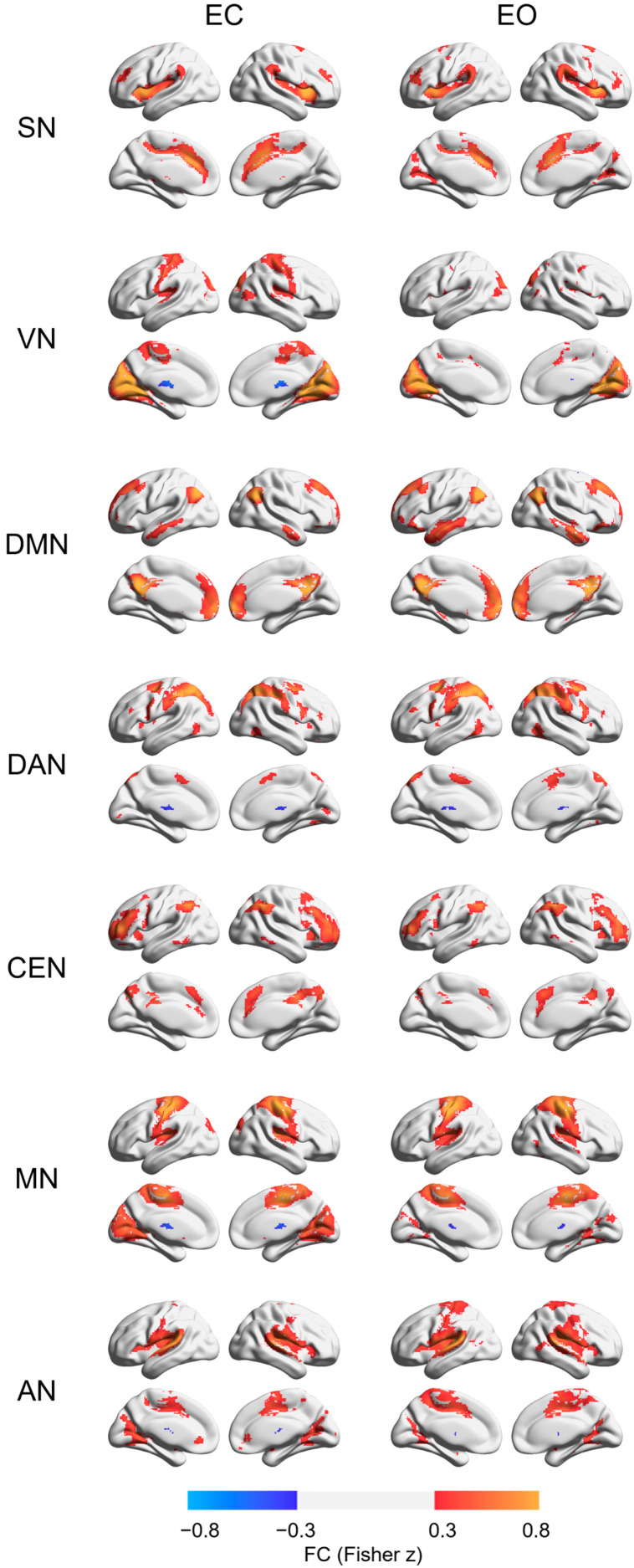
Seven FC maps of networks in EC and EO conditions based on New Taipei City dataset. The left and right column displays the FC maps of each network in EC and EO conditions, respectively. The color range of FC maps represents the Fisher z value from −0.8 to −0.3 and from 0.3 to 0.8.

Based on our two datasets, one sample t-test of FC identified seven resting networks for both EC and EO conditions, respectively. Figure 1 depicts the seed regions of the seven RSNs and their FC maps during EC and EO conditions based on the combined dataset. Seven RSNs during EC and EO conditions based on each separate dataset are also shown in Figure A1 (for Berlin dataset) and Figure A2 (for New Taipei City dataset), which showed similar findings between the two datasets. These seven RSNs are: SN, VN, DMN, DAN, CEN, MN and AN (Fisher z > 0.3 and <−0.3).

**Figure 2:**
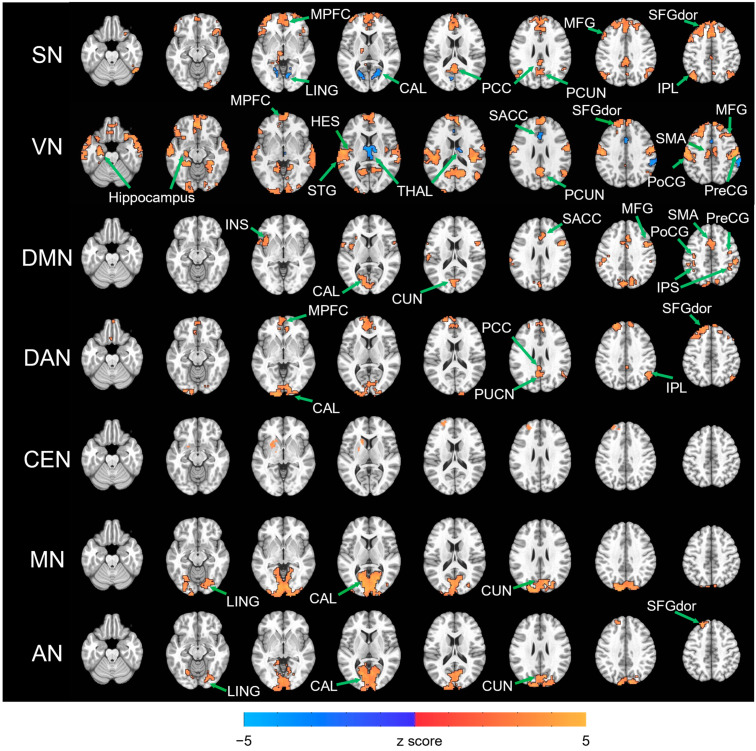
Paired *t*-test of FC difference (EC–EO) for each resting state network. All resulting statistical map were thresholded at voxel-wise *p* < 0.005 and cluster-wise alpha < 0.05. The regions, which showed significant difference and were identified to one of the seven networks, were marked with their names. For each network, eight slices are at z = −23, −13, −3, 7, 17, 27, 37, 47, respectively.

For each network, paired *t*-tests comparing EC and EO revealed several regions evincing significant FC differences. All resulting statistical maps were thresholded at voxel-wise *p* < 0.005 and cluster-wise alpha < 0.05 and are displayed in Figure 2. Regional details of these results are described in Table 2.

**Figure 3:**
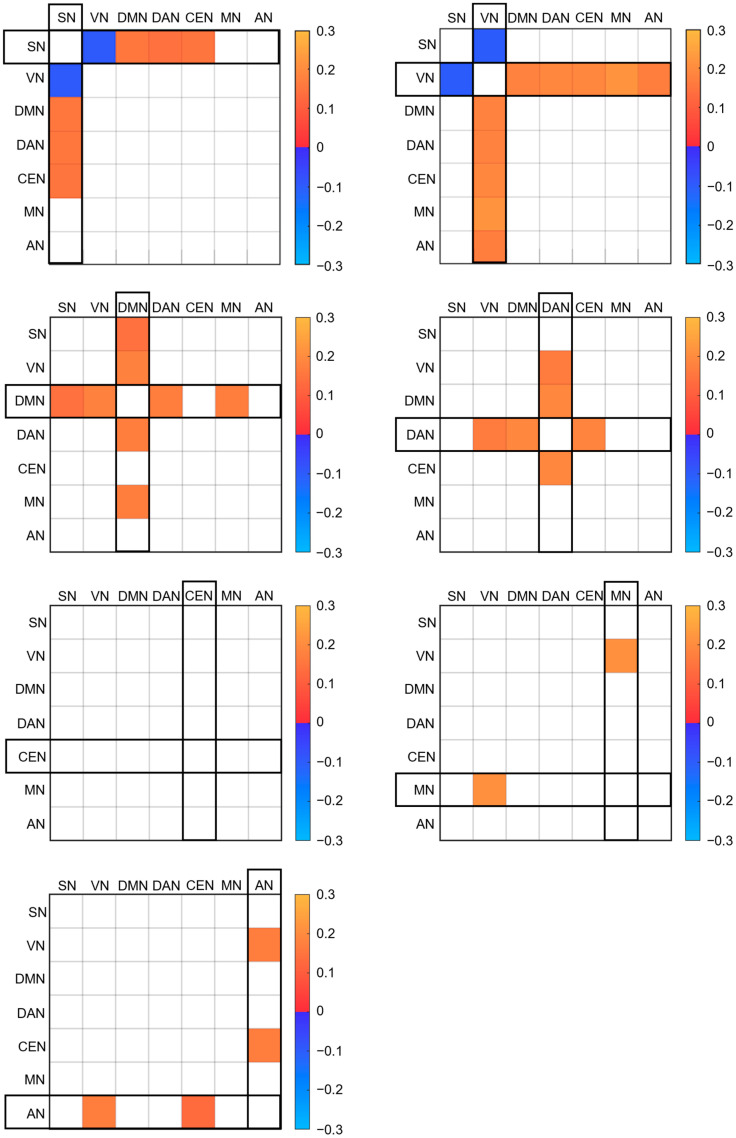
The FC differential patterns (EC–EO) between networks (*p* < 0.05, FDR corrected). Red colored boxes indicate higher FC, while blue colored ones indicate lower FC during EC. The black rectangular box indicates which networks we selected for comparison to others within the matrices. The color range represents the inter-network FC difference (Fisher z).

Furthermore, we examined each region evincing FC differences and identified to which network they belong. All the identified regions which belong to one of the seven networks are marked in Figure 2 and all of these FC differential patterns between networks are depicted in Figure 3 (*p* < 0.05, FDR corrected). In Figure 3, matrices show the FC difference of each network with the other six networks. Red colored boxes indicate higher FC, while blue colored ones indicate lower FC during EC. The black rectangular box indicates which networks we selected for comparison to others within the matrices.

In brief, there were three main findings in this study: (1) more increased connectivity among RSNs were found under EC than EO conditions. (2) Three sensory networks, including AN, MN and VN showed increased FC with each other during EC. (3) SN evinced increased FC with DMN and decreased FC with VN when shifting from EO to EC.

First of all, more RSNs evinced increased FC with each other during EC than EO. For SN, increased FC with DMN (MPFC, PCC and IPL), DAN (MFG), CEN (SFGdor, PCUN and IPL) were found. For VN, increased FC with DMN (MPFC), DAN (MFG), CEN (SFGdor and PCUN), MN (PreCG, PoCG and SMA) and AN (HES and STG) were found. For DMN, increased FC with SN (SACC and INS), VN (CAL, CUN), DAN (MFG and IPS) and MN (PreCG, PoCG and SMA) were found. For DAN, DMN (MPFC, PCC and IPL), VN (CAL) and CEN (SFGdor, PCUN and IPL) exhibited higher FC. For MN, increased FC with VN (CAL, CUN and LING) were found. For AN, increased FC with VN (CAL, CUN and LING) and CEN (SFGdor) were found.

Moreover, the sensory networks (AN, MN and VN) evinced increased FC with one another in EC compared to EO. For VN, we found an increase in FC with MN and VN. For MN, higher FC was found with VN. For AN, increased FC was found with VN.

Last but not least, when changing from EO to EC, SN was found as a distinctive network that evincing increased FC with DMN and decreased FC with VN. For SN, reduced FC with VN (CAL and LING) and higher FC with DMN were found during EC. For VN, decreased FC with SN (THAL, SACC) was found. For DMN, increased FC with SN (SACC and INS) was also found.

## 4. Discussion

In this study, a voxel-wise FC analysis was conducted to explore variant connectivity patterns among RSNs, comparing EC and EO conditions. Our main findings are as follow: (1) More RSNs evinced increased connectivity with one another under EC than under EO conditions. (2) Three sensory networks (AN, MN and VN) evinced increased connectivity with one another during EC. (3) SN was distinctive among the networks in that, when changing from EO to EC, it evinced increased connectivity with DMN and decreased connectivity with VN. Overall, our results suggested that most RSNs exhibited stronger correlations during EC, by comparison to EO.

Previous studies have claimed that there are two information processing modes in the brain: an integrated processing mode and a specialized processing mode [26]. Graph theory estimates suggest that cliquishness and local efficiency of networks were lower, but global efficiency was higher during EC, compared to EO. This finding might suggest that, during EC, there is an increase in integrated information processing, along with a decrease in specialized information processing [7]. In addition, during EC, higher FC is evinced among regions [5,6,27] and RSNs [4]. What is more, during EO, FC within DMN increased, perhaps implying that processing is more specialized under the EO condition [8]. Our findings are consistent with these lines of research that suggest a shift in information processing mode—RSNs evince greater integration during EC and greater specialization during EC.

Specifically, the present study evinced a stronger association among the sensory networks VN, MN and AN; this association indicates a higher coupling activation of sensory modalities during EC. Two states of perceptual brain activity have been proposed before: the “exteroceptive” state, characterized by attention and ocular motor activity during EO and the “interoceptive” state, characterized by imagination and multisensory activity during EC [28]. As regards our findings, we found elevated FC between VN and the hippocampus (Figure 2), which might contribute to recall of sensory experiences. In other words, these lines of evidence converge in suggesting that co-activation of multiple sensory networks during EC facilitates imaginative recall of sensory experiences [28].

An especially interesting finding of our investigation is the evidence of higher SN-DMN connectivity during EC; this suggests a high degree of synchrony between SN and DMN. Previously, SN was found to play an important role in switching between DMN and CEN [29,30]. Von Economo neurons (VENs), which have only been found within SN [31], have been proposed to function as a mechanism for relaying output signals to other parts of the brain [32]. Our findings are consistent with this proposal in suggesting that SN facilitates the transfer of signals to different parts of the brain.

Concerning DMN, previous research has indicated that it is involved in internal cognitive activities, including daydreaming or mind-wandering [33,34], self-related thought [35,36,37], autobiographical memory [38,39], self-reference [35,40], cognitive mapping [41,42] and imaginative scene construction [43]. Importantly, other lines of research have indicated that SN modulates activation of DMN when transitioning from social interactive tasks to non-social tasks [44], from task to rest [45], or from EC to EO [46] and indeed that DMN and SN are anatomically connected [47]. When these findings are combined with the results reported here, the higher synchronization between SN and DMN that we found might imply that there could be a signal transmission pathway from SN to DMN. In virtue of the higher connectivity of multiple sensory networks evinced during EC, we infer that this conjectured signal pathway from SN to DMN may facilitate the brain’s devoting more attention to internal information processing during EC as, for example, with the recall of sensory experience.

On the other hand, the increased connectivity between SN and VN under the EO condition suggests enhancement of the coupling signal between SN and VN. Previous studies indicated that local neuronal activity in SN and VN increased concurrently. Moreover, the spatial pattern of increased SN-VN FC coincided almost exactly with the pattern of increased neuronal activity [48].

Intriguingly, it has been proposed that a cortical feedback loop modulates activity in the visual cortex [49] and that, during EO, top-down signaling from the SN modulates the VN’s primary visual cortex [49,50]. Consistent with these studies, our results evinced greater synchronization between SN and VN during EO, by comparison to EC. Given that during EO exteroceptive activity characterized by attention and ocular motor activity is enhanced [28], it might be that visual input might raise our level of attention or alertness, thereby suggesting increased SN local activity. The increase in attention or alertness might in turn might then enhance the modulation of VN by SN.

Previous studies suggested that SN was found to be effective at discriminating between the EC and EO resting conditions [51] and that it mediates interaction with other large-scale networks that are engaged during transitions between externally oriented attention and internally oriented self-related mental process [50]. Our findings show that only SN evinced increased connectivity with DMN and decreased connectivity with VN, during EC and relative to EO. This distinctive pattern differs from other networks and adds further confirmation to the conjecture that among SN’s functions is discrimination between EC and EO resting states. In view of the evidence about the patterns of connectivity among SN, DMN and VN, we infer that SN might function in a manner analogous to a circuit switch for DMN and VN activation, triggered by behavioral transitions between EC and EO. In this way SN would be driving a stronger modulation of DMN for internally oriented, self-related processing, while attenuating its effects on VN (for externally oriented attention) during EC. The reverse then obtains for EO. This might help to explain why intrinsic self-related neural processing is more readily tracked in the EC than in the EO condition [52].

Functional connectivity analysis, however, cannot give direct evidence concerning the hypothesized causal connectedness that seems to link SN to DMN and to VN, differentially, for EO and EC conditions. Therefore, in the future, we would like to use dynamic causal modelling, DCM [53] in order to assess what model is best suited to describing causal connectedness for these two resting state conditions. First, we would test for EC/EO differences in signal derivation for the three networks. Second, using Bayesian model selection, we would compare these potential models to select the optimal one for each resting state [54]. And third, we would use diffusion tensor imaging (DTI) to detect differences in anatomical connectivity among SN, DMN and VN, for EC and EO conditions.

## 5. Conclusions

In sum, EC and EO behavioral changes during the resting state modifies connectivity among RSNs. By assessing the difference of FC among seven RSNs for both EC and EO, we revealed patterns of variation in the amount and intensity of connectivity among RSNs that suggest distinct modes of information processing. EC tends to correlate with greater integration; EO, with greater specialization. Furthermore, three sensory networks (viz. VN, MN and AN) were found to evince heightened connectivity during EC, which may correlate with the ability to recall sensory experiences. Finally, when shifting from EO to EC, SN was found to evince more connectivity with DMN and less with VN. In view of previous, related research, we conjecture that a function of SN is to discriminate between two resting conditions that differentially affect DMN and VN. It might be the case that SN functions in a manner analogous to a circuit switch, modulating DMN and VN, driven by behavioral between EC and EO.

## Figures and Tables

**Table 1:** Seed regions selected for each resting state network.

Regions	Location	Coordinate (MNI)			References
		X	Y	Z	
**SN**					[20]
SACC	M	−1	20	28	
AI	L	−42	14	0	
AI	R	40	12	0	
THAL	L	−12	−16	4	
THAL	R	14	−20	8	
**VN**					[21]
PVC	R	20	−66	2	
PVC	L	−20	−66	2	
**DMN**					[20]
PCC	M	0	−46	20	
MPFC	M	0	56	−6	
IPL	L	−42	−68	38	
IPL	R	50	−60	36	
**DAN**					[21]
pIPS	L	−26	−65	52	
pIPS	R	28	−65	51	
FEF	L	−29	−5	55	
FEF	R	31	−5	54	
aLPS	R	43	−36	46	
aLPS	L	−45	−37	48	
**CEN**					[20]
DLPFC	L	−36	52	10	
DLPFC	R	34	46	20	
IPL	L	−40	−56	44	
IPL	R	46	−52	44	
PCUN	M	4	−42	44	
**MN**					[22]
MC	L	−40	−23	53	
MC	R	41	−22	48	
SMA	M	1	−18	49	
**AN**					[22]
PAC	L	−64	−28	13	
PAC	R	62	−24	13	

The sizes of all seed regions were 6 mm radius.

**Table 2:** Regional details of FC difference for each network.

RSN	Cluster (Voxel)	Peak Coordinate (MNI)			Regions within Cluster
		X	Y	Z	
SN	EC > EO				
	1523	1	25	52	MPFC, MFG, SFGdor, MCC, SACC
	384	−2	−62	25	PCC, PCUN
	230	−41	58	4	MFG, SFGdor
	227	−41	−74	49	ANG
	164	55	−59	−23	ITG, IOG
	137	46	−65	49	IPL, ANG
	136	49	25	−8	IFGord
	EO > EC				
	134	22	−59	4	CAL, LING
	105	−8	−77	16	CAL, LING
VN	EC > EO				
	4713	−56	−23	55	PreCG, PoCG, SMA, HES, STG
	1573	−8	49	−11	MPFC, SMA, SFGdor, MFG
	943	13	−65	19	PCUN, CUN
	253	−20	−104	−2	CAL
	251	−53	13	−26	MTP
	174	10	−89	−5	CAL
	152	49	−59	25	ANG
	98	−23	−17	−20	Hippocampus
	EO > EC				
	161	13	−11	13	THAL
	107	1	31	22	SACC
	96	61	−35	46	SMG
DMN	EC > EO				
	459	−14	−80	10	CAL, CUN
	342	−38	−56	61	IPS, PreCG, PoCG
	313	1	7	46	SMA, SACC, MCC
	163	−41	1	7	INS, ROL, TPOsup
	152	31	−56	55	IPS, SMG
	143	49	7	28	PreCG, IFGoperc
	119	49	−5	52	PreCG, MFG
DAN	EC > EO				
	1129	−11	40	52	MPFC, SFGdor, MFG
	513	−14	−107	−5	CAL, IOG
	135	−8	−56	28	PCC, PCUN
	116	49	−59	37	IPL, ANG
CEN	EC > EO				
	118	−17	19	−2	CAU, PUT
	109	−23	49	40	SFGdor
MN	EC > EO				
	2487	13	−101	5	CAL, CUN, LING
AN	EC > EO				
	1061	−11	−86	4	CAL, CUN, LING
	110	−11	43	49	SFGdor

Threshold of significance: voxel-wise *p* < 0.005, cluster-wise alpha < 0.05. Size of voxel = 3 mm^3^. MCC = middle cingulate; ANG = angular; ITG = inferior temporal gyrus; IOG = inferior occipital gyrus; IFGord = inferior frontal gyrus pars orbitalis; MTP = medial temporal pole; SMG = supramarginal gyrus; ROL = rolandic operculum; TPOsup = superior temporal pole; IFGoperc = opercular inferior frontal gyrus; CAU = caudate nucleus; PUT = putamen.

## Data Availability

The data that support the findings of this study are available on request from the corresponding author. The data are not publicly available due to privacy restrictions.
